# Preliminary Evidence That CD38 Moderates the Association of Neuroticism on Amygdala-Subgenual Cingulate Connectivity

**DOI:** 10.3389/fnins.2020.00011

**Published:** 2020-02-14

**Authors:** Benjamin A. Tabak, Katherine S. Young, Jared B. Torre, Baldwin M. Way, Lisa J. Burklund, Naomi I. Eisenberger, Matthew D. Lieberman, Michelle G. Craske

**Affiliations:** ^1^Department of Psychology, Southern Methodist University, Dallas, TX, United States; ^2^Social, Genetic and Developmental Psychiatry Centre, Institute of Psychiatry, Psychology and Neuroscience, King’s College London, London, United Kingdom; ^3^Department of Psychology, University of California, Los Angeles, Los Angeles, CA, United States; ^4^Department of Psychology, The Ohio State University, Columbus, OH, United States

**Keywords:** CD38, fMRI, functional connectivity, neuroticism, psychopathology, oxytocin

## Abstract

CD38 genetic variation has been associated with autism spectrum disorders and social anxiety disorder, which may result from CD38’s regulation of oxytocin secretion. Converging evidence has found that the rs3796863 A-allele contributes to increased social sensitivity compared to the CC genotype. The current study examined the moderating role of CD38 genetic variants (rs3796863 and rs6449182) that have been associated with enhanced (or reduced) social sensitivity on neural activation related to neuroticism, which is commonly elevated in individuals with social anxiety and depression. Adults (*n* = 72) with varying levels of social anxiety and depression provided biological samples for DNA extraction, completed a measure of neuroticism, and participated in a standardized emotion processing task (affect matching) while undergoing fMRI. A significant interaction effect was found for rs3796863 x neuroticism that predicted right amygdala-subgenual anterior cingulate cortex (sgACC) functional connectivity. Simple slopes analyses showed a positive association between neuroticism and right amygdala-sgACC connectivity among rs3796863 A-allele carriers. Findings suggest that the more socially sensitive rs3796863 A-allele may partially explain the relationship between a known risk factor (i.e. neuroticism) and promising biomarker (i.e. amygdala-sgACC connectivity) in the development and maintenance of social anxiety and depression.

## Introduction

The multifunctional protein CD38 (Cluster of Differentiation 38) contributes to individual differences in social cognition and behavior, which may result from CD38’s regulation of oxytocin secretion (Jin et al., 2007). The majority of human research associating CD38 genetic variation and social phenotypes has focused on two genetic variants of interest, rs3796863 (located in intron 7 on chromosome 4p15; Malavasi et al., 2008), and rs6449182 (located in a regulatory region in intron 1; Ferrero et al., 1999). Compared to individuals with the rs3796863 CC genotype, A-allele carriers have been associated with enhanced social sensitivity in the form of increased parental sensitivity (Feldman et al., 2012), higher levels of empathy and altruism (Liu et al., 2017), and decreased risk of social impairments and autism spectrum disorders (Lerer et al., 2010; Munesue et al., 2010). Individuals carrying the A-allele have shown greater CD38 gene expression (Lerer et al., 2010) and higher levels of unextracted plasma oxytocin (Feldman et al., 2012) in comparison to individuals with the CC genotype. However, contrary to previous results demonstrating beneficial socioemotional outcomes associated with the rs3796863 A-allele, our research group found that among individuals who experienced higher levels of interpersonal stress, A-allele carriers had *higher* levels of social anxiety and depression over a 6-year period compared to those with the CC genotype (Tabak et al., 2016).

As research on oxytocin (and related genes such as CD38), has progressed, paradoxical results such as these have led to the hypothesis that oxytocin enhances sensitivity to positive *or* negative social stimuli (Olff et al., 2013; Shamay-Tsoory and Abu-Akel, 2015). Work focusing on oxytocin system genes has shown that variants associated with enhanced social sensitivity may contribute to positive or negative outcomes depending on relevant environmental factors and individual differences (Tabak, 2013). For example, several studies focused on variation in the oxytocin receptor gene polymorphism rs53576 have found that G-allele carriers who experienced childhood maltreatment were at greater risk for mental health concerns (Bradley et al., 2011; McQuaid et al., 2013; Andreou et al., 2018), even though the majority of research examining this SNP has found the G-allele to be beneficial or protective. Further research focusing on variations in oxytocin system genes has shown that alleles previously associated with beneficial social outcomes may also be related to psychopathology when accounting for relevant moderators (Kushner et al., 2018). Together, studies such as these demonstrate that variation in oxytocin system genes, including CD38, may contribute to enhanced levels of social sensitivity, which can exacerbate the effects of environmental stressors that contribute to the development and maintenance of psychopathology (Tabak, 2013). This is particularly relevant because positive associations between oxytocin and human social processes have often overshadowed evidence of the potential role of oxytocin in the development of psychopathology (McQuaid et al., 2014).

In the present study, we sought to build on our previous findings (Tabak et al., 2016) by investigating the underlying mechanisms that connect CD38, social sensitivity, and psychopathology. To examine this question, we focused on how CD38 genetic variation moderated a neural circuit that includes regions that have been associated with hyperactivation in both depression and social anxiety; specifically, we examined connectivity between the subgenual anterior cingulate cortex (sgACC) and the amygdala.

A host of neuroimaging research has focused on the sgACC and amygdala in depressed individuals (for review see Ressler and Mayberg, 2007). There is evidence of heightened activation in the amygdala and sgACC in individuals with depression when viewing negative stimuli, and post-treatment decreases in depression symptoms have been associated with decreased activation in these regions (Ressler and Mayberg, 2007). Studies have also confirmed connectivity between the amygdala and sgACC (Stein et al., 2007) and this neural circuit has important relevance for emotion dysregulation, a prominent characteristic of mood disorders (Joormann and Vanderlind, 2014). Findings have shown greater positive amygdala-sgACC functional connectivity in depressed adolescents during resting-state (Connolly et al., 2013) and while processing fearful facial stimuli (Ho et al., 2014) compared to healthy controls. Similar results have emerged in relatives of individuals diagnosed with major depressive disorder (Wackerhagen et al., 2017). Studies of individuals with social anxiety disorder have also found increased amygdala activation during emotional face processing (Ball et al., 2012) and when viewing negative (e.g. fearful or threatening) stimuli compared to healthy controls (Freitas-Ferrari et al., 2010; Gentili et al., 2016). In addition, meta-analytic effects for increased activation in the sgACC have been found in individuals with social anxiety disorder (Gentili et al., 2016). Thus, there is evidence for amygdala and sgACC hyperactivation in both depression and social anxiety disorder, and evidence for altered functional connectivity between these regions in depression.

Elevated levels of neuroticism are a risk factor for depression and anxiety, including social anxiety (Kotov et al., 2010). Therefore, neuroticism is often examined as a trait level individual difference that is positively associated with current levels of anxiety and depression, as well as potentially higher future levels of psychopathology. Neuroticism is also associated with more negative responses to stress, increased reactivity to threatening stimuli (Barlow et al., 2014), and heightened activation in the amygdala and sgACC (Haas et al., 2007). Given the relationship between neuroticism, psychopathology, and threat reactivity, it is important to note that a meta-analysis of neuroimaging studies examining neuroticism and emotion processing did not find an association between neuroticism and amygdala activation (Servaas et al., 2013). Rather, findings from Servaas et al. (2013) suggest that the role of neuroticism in amygdala activation appears to be related to altered connectivity between the amygdala and frontal regions that result in emotion dysregulation (Servaas et al., 2013). Indeed, Cremers et al. (2010) found more inverse functional connectivity in the left amygdala and anterior cingulate cortex among individuals with higher levels of neuroticism when viewing negative stimuli. Previous work by Pezawas et al. (2005) also found that inverse connectivity between the amygdala and sgACC was associated with increased harm avoidance (a construct highly correlated with neuroticism that has been associated with affective disorder symptomology; Jylhä and Isometsä, 2006) in short allele carriers in the 5-HTTLPR polymorphism. In sum, previous findings suggest that higher levels of neuroticism and altered connectivity between the amygdala and sgACC may represent a common neurobiological mechanism underlying the development of social anxiety disorder and major depression.

In the present study, based on the associations between CD38 genetic variation and affective reactivity (Sauer et al., 2012), social anxiety, and depression (Tabak et al., 2016), we examined the relationship between amygdala-sgACC connectivity and neuroticism in individuals with varying levels of social anxiety and depression. Using an *a priori* seed-based approach, we used psychophysiological interaction (PPI) analysis to investigate whether CD38 moderates the relationship between neuroticism and amygdala-sgACC connectivity. We hypothesized that higher levels of neuroticism would be related to positive connectivity in this neural circuit in individuals with genotypes (i.e. the rs3796863 A-allele) that have been associated previously with enhanced social sensitivity. We also examined variation in a second CD38 SNP, rs6449182, since there is evidence that this polymorphism is functional and the G allele is associated with increased CD38 expression (Jamroziak et al., 2009; Polzonetti et al., 2012; but see Riebold et al., 2011).

## Methods

### Participants

The present study includes a subsample from a randomized controlled trial examining the effectiveness of two types of psychotherapy for social anxiety disorder plus a healthy control comparison group (see Craske et al., 2014 for full methods). The current study focused on measurements obtained at baseline before any intervention began and included participants who provided a saliva sample for genotyping and fMRI data (*n* = 81). Therefore, methods refer to only this aspect of the study for these participants. Participants were 18–45 years old, right-handed, and English speaking. They were either free of medications, or stabilized on medication, and were not currently involved in behavioral therapy (see Craske et al., 2014 for full exclusion criteria).

No genotype could be determined for three participants and six participants’ fMRI data were removed due to high levels of motion-induced noise (>10% of images had a global signal intensity >2.5 SD of mean, or were affected by motion of >2.5 mm in any direction; Young et al., 2017). This resulted in 72 participants (39 male; 33 female; *Mean age* = 27.56; *Age range* = 18–43). Participants self-identified as Caucasian (45.8%), Asian American (25%), Hispanic (13.9%), and Other (15.3%). This study was carried out in accordance with the recommendations of the UCLA Office for the Protection of Human Research Subjects and approved by the UCLA Institutional Review Board. All participants provided written informed consent in accordance with the Declaration of Helsinki.

### Materials

#### Neuroticism

The 12-item neuroticism subscale of the Eysenck Personality Questionnaire–Revised Short form (EPQR–S; Eysenck et al., 1985) was used to measure neuroticism (α = 0.86).

#### Psychiatric Diagnosis

Even though we focused on trait levels of neuroticism, the majority of participants (*n* = 57) met diagnostic criteria for social anxiety disorder. Fifteen additional participants did not meet criteria for any diagnosis (i.e. they were a healthy control comparison group). Diagnoses were based on the Diagnostic and Statistical Manual of Mental Disorders, 4th Edition through the use of the Anxiety Disorders Interview Schedule-IV (Brown et al., 1994) that were conducted by trained interviewers. Individuals who met criteria for a clinical disorder all had a current diagnosis of social anxiety disorder that was either principal or co-principal, with a clinical severity rating of four or higher (Craske et al., 2014). Healthy controls did not have a current or previous psychiatric diagnosis. Among participants who met criteria for social anxiety disorder, 13.9% (rs3796863 CC *n* = 7, A carrier *n* = 3; rs6449182 CC *n* = 8, G carrier *n* = 2) were currently taking medication for anxiety, depression, or “another emotional problem” (see Burklund et al., 2015 for additional details).

#### Genotyping

Participants provided saliva samples using Salivettes (Sarstedt, Germany). DNA Extraction and genotyping was performed by Genomeadvisors Inc., La Mirada, CA, United States. CD38 SNPs were genotyped using Taqman SNP Genotyping Assays (rs6449182: C___1216863_10; rs3796863: C___1216944_10) with the ABI 7900 Sequence Detection System.

### Procedure

The EPQR-S was administered 1–2 weeks before participants completed their fMRI session. Before beginning the fMRI procedure, participants practiced the reactivity task that involved viewing and matching images of emotional facial expressions and geometric shapes (Hariri et al., 2002). In the present study, our interest was in examining neural reactivity to negative stimuli (angry, disgusted, or fearful emotional expressions) obtained from the NimStim Face Stimulus set (Tottenham et al., 2009). We collapsed across facial expressions in analyses to examine responses to negative facial expressions in general compared to shape matching. This resulted in two conditions: affect match and shape match. Our focus of analysis was on the contrast between matching affect vs. matching shapes, which is a well-validated method of assessing neural activation associated with viewing emotionally evocative human stimuli while controlling for attention and motoric responses (as described in Burklund et al., 2015). This task has been used in previous research examining amygdala-sgACC functional connectivity and depression (Pezawas et al., 2005). Participants also completed two other conditions in which they were asked to engage in affect labeling or gender labeling of the face stimuli (see Burklund et al., 2015 for further details). Regressors for these stimuli were included in first level models, but as they are not the focus of the current investigation, they are not reported on here. A previous study by our research group (Burklund et al., 2015) also examined neural activation across different clinical subgroups compared to healthy controls in the bilateral amygdala as well as right ventral lateral prefrontal cortex during affect match vs. shape match. In contrast, the current study examined trait levels of neuroticism and focused on functional connectivity between the amygdala and sgACC.

As described by Burklund et al. (2015) we used a block design for stimuli presentation with four blocks per condition (affect match, shape match, affect label, gender label; all conditions were counterbalanced) and six trials per block (trials lasted 5 s, resulting in 30 s blocks). Preceding the stimuli blocks were 10 s fixation crosshairs and 3 s instruction cues. The present analyses build on the prior work published in Burklund et al. (2015) by examining genetic contributions to functional connectivity between areas as a function of neuroticism rather than focusing on group differences in neural activation as was done in the prior work. A Macintosh MacBook Pro computer with MacStim software (WhiteAnt Occasional Publishing)^1^ and high-resolution goggles (Resonance Technology, Inc.) were used to present stimuli. Responses were collected with an fMRI-compatible button box through a custom USB interface.

### fMRI Image Acquisition

Magnetic resonance images were acquired using a Trio 3.0 Tesla Siemens MRI scanner at the UCLA Ahmanson-Lovelace Brain Mapping Center. For each participant, a high-resolution structural T2-weighted echoplanar imaging volume (spin-echo, TR = 5000 ms, TE = 34 ms, matrix size = 128 × 128, resolution = 1.6 mm × 1.6 mm × 3 mm, FOV = 200 mm, 36 slices, 3 mm thick, flip angle = 90°, bandwidth = 1302 Hz/Px) was acquired coplanar with the functional scans. Four functional runs were acquired, with a total of 344 volumes (gradient-echo, TR = 3000 ms, TE = 25 ms, flip angle = 90°, matrix size = 64 × 64, resolution = 3.1 mm x 3.1 mm x 3.0 mm, FOV = 200 mm, 36 axial slices, 3 mm thick, bandwidth = 2604 Hz/Px).

### fMRI Pre-processing and Analysis

Imaging data were analyzed using SPM8 (Wellcome Trust Centre for Neuroimaging, University College London, United Kingdom)^2^. Functional images for each participant were realigned to correct for head motion, co-registered to the high-resolution structural images, normalized into a standard stereotactic space as defined by the Montreal Neurological Institute and smoothed with an 8 mm Gaussian kernel FWHM. Experimental blocks were modeled using a boxcar function convolved with the canonical hemodynamic response. Motion parameters were included in the model as regressors of no interest. Linear contrasts for affect match vs. shape match were computed at the first-level for each participant using a fixed-effects model. PPI analyses (Friston et al., 1997) were implemented using generalized PPI (gPPI) within SPM8 (McLaren et al., 2012). These analyses were used to examine whether the interaction between neuroticism and CD38 variation predicted functional connectivity between the amygdala and the sgACC. The right and left amygdala were used as separate seed regions for these analyses [anatomically defined ROI; Automated Anatomical Labeling (AAL) library]. We conducted both an ROI-based analysis and a whole-brain analysis to investigate general alterations in right and left amygdala connectivity, focusing on the sgACC. A spherical sgACC ROI (6 mm radius) was created based on coordinates in a previous study examining the moderating role of genetic variation on amygdala-sgACC connectivity during the same affect match task used in the present study (Pezawas et al., 2005; MNI coordinates: *x* = 0, *y* = 37, *z* = -2).

### Statistical Analysis

ROI analyses: All continuous independent variables and covariates were mean centered before analyses. Using hierarchical multiple linear regression, separate analyses were conducted for each CD38 SNP that included the following predictors of right (or left) amygdala-sgACC connectivity: (a) the main effect of genotype, (b) the main effect of neuroticism, and (c) the interaction effect of genotype x neuroticism. Following the recommendations of Keller (2014) we also ran analyses with the inclusion of additional covariates to assess the robustness of findings including: self-reported race/ethnicity (Asian, Hispanic, Other; Caucasians were designated as the comparison group), gender, age, medication status, group (i.e. clinical vs. healthy controls), and all genotype x covariate as well as neuroticism x covariate interactions. The addition of all robustness covariates and their interactions did not alter the significance of any primary interaction effects.

**FIGURE 1:**
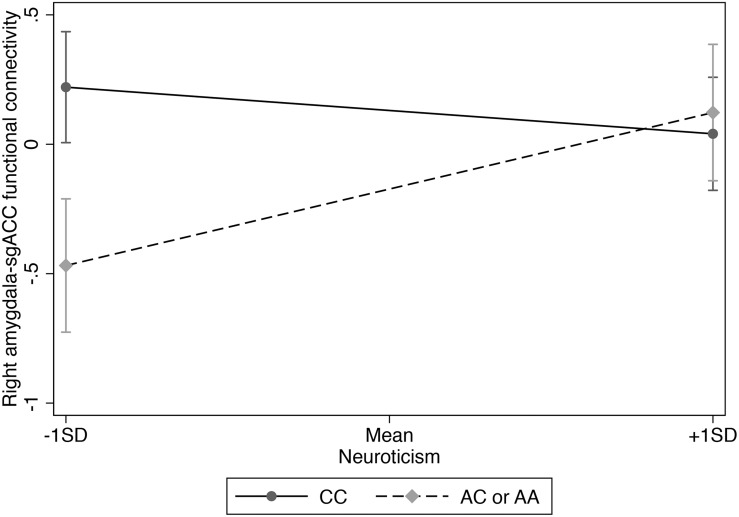
Interaction of rs3796863 x right amygdala-sgACC functional connectivity predicting neuroticism.

Significant interactions were followed by simple slopes analyses to examine the main effects of neuroticism for each genotype group. Analyses were conducted using SPSS 24 and the PROCESS macro (Hayes and Little, 2017). Figure 1 was created using Stata version 14. Bonferroni correction was used to correct for multiple testing for the four primary gene x neuroticism tests (i.e. rs3796863 x neuroticism for left and then right amygdala, and the same two tests for rs6449182), resulting in a threshold of *p* < 0.0125.

As in previous studies (Feldman et al., 2012; Sauer et al., 2012; Tabak et al., 2016), we used dominant coding for rs3796863 (CC = 0; A-allele carriers [AC and AA] = 1). Based on previous work (Jamroziak et al., 2009; Polzonetti et al., 2012), rs6449182 was also coded in a dominant manner (CC = 0; G-allele carriers [CG or GG] = 1). Genotype frequencies for the total sample of participants who provided genetic and fMRI data were in Hardy–Weinberg Equilibrium (rs3796863: χ^2^ = 2.6, *p* = 0.11, rs6449182: χ^2^ = 2.4, *p* = 0.12).

Whole brain analyses: Group level whole brain multiple regression analyses were conducted, entering connectivity SPM images for the contrast “Affect Match – Shape Match.” Regressors included in the model were the CD38 genotype, neuroticism, and CD38 x neuroticism interaction effects. Gender, age, race/ethnicity, medication status, group, and all genotype x covariate as well as neuroticism x covariate interactions were entered as covariates of no interest.

## Results

Table 1 displays sample demographics, means, standard deviations, and genotype frequencies. No significant differences in demographic variables were found between dichotomized genotype groups. Our interest in focusing on neuroticism as a trait level individual difference that reflects anxiety and depression symptoms was confirmed by high correlations (*rs* = 0.73) between neuroticism and the General Distress Anxiety and Depression scales from the Mood and Anxiety Symptoms Questionnaire (Watson et al., 1995). We first examined the correlation between CD38 genotype and neuroticism (including gender, age, race/ethnicity, medication status, and group as covariates) and found no associations between rs3796863 genotype (*A/C or A/A* genotypes coded 1; *C/C* genotype coded 0) (*r* = 0.02, *p* = 0.88) or rs6449182 genotype and neuroticism (*G/G or C/G* genotypes coded 1; *C/C* genotype coded 0) (*r* = 0.05, *p* = 0.71).

**TABLE 1:** Descriptive statistics for rs3796863, rs6449182, and major study variables.

**Variable**	**All participants**	**rs3796863 A-Allele Carriers**	**rs3796863 CC Homozygotes**	**rs6449182 G-Allele Carriers**	**rs6449182 CC Homozygotes**
*Gender*		*t* = −1.34 (70)		*t* = −0.478 (69)	
Female	34(47.2%)	16(57.1%)	18(40.9%)	10(52.6%)	24(46.2%)
Male	38(52.8%)	12(42.9%)	26(59.1%)	9(47.4%)	28(53.8%)
		*t* = 1.44 (58.5)		*t* = −1.22 (69)	
Age	27.56 (6.51)	26.21 (6.33)	28.44 (6.54)	29.14 (7.13)	26.94 (6.3)
		*t* = 0.156 (70)		*t* = −0.101 (69)	
Neuroticism	6.83 (3.59)	6.75 (3.72)	6.89 (3.54)	7.00 (2.85)	6.90 (3.76)
*Race/ethnicity*		χ^2^ = 2.53 (3, 72)		χ^2^ = 2.02 (3, 71)	
Caucasian	33(45.8%)	15(53.6%)	18(40.9%)	10(52.6%)	22(42.3%)
Hispanic/Latino	10(13.9%)	2(7.1%)	8(18.2%)	2(10.5%)	8(15.4%)
Asian American/Pacific Islander	18(25%)	6(21.4%)	12(27.3%)	3(15.8%)	15(28.8%)
Other	11(15.3%)	5(17.9%)	6(13.7%)	4(21.1%)	7(13.5%)
*CD38 genotype*					
AA	8(11.1%)	–	–	–	–
AC	20(27.8%)	–	–	–	–
CC	44(61.81%)	–	–		–

### CD38 Variant rs3796863

We used hierarchical multiple linear regression analysis and found a main effect of CD38 rs3796863 genotype on right amygdala-sgACC functional connectivity (*p* = 0.017), but no main effect of neuroticism (see Table 2). However, there was also a significant rs3796863 x neuroticism effect (*p* = 0.002) that maintained significance following multiple test correction. As shown in Table 2 and Figure 1, simple slopes analysis showed a positive association between neuroticism and right amygdala-sgACC connectivity for A-allele carriers, but the simple slope for individuals with the CC genotype was not significant. There were also no main or interaction effects of genotype or neuroticism when examining left amygdala-sgACC connectivity (See Table 2).

**TABLE 2:** **(a)** CD38 rs3796863 and neuroticism predicting right amygdala-sgACC functional connectivity. **(b)** CD38 rs3796863 and neuroticism predicting left amygdala-sgACC functional connectivity.

**Independent variable**	***b***	**β**	***SE***	***R*^2^**
**(a)**				
CD38 genotype	−0.312*	–0.282	0.128	0.066
Neuroticism	0.020	0.132	0.018	0.097
Genotype x Neuroticism	0.107**	0.798	0.033	0.217
*Simple Slope for A-allele carriers*				
Neuroticism	0.082**	0.608	0.021	0.370
*Simple Slope for C/C genotype*				
Neuroticism	–0.025	–0.162	0.024	0.026
**(b)**				
CD38 genotype	0.005	0.005	0.125	0.000
Neuroticism	–0.003	–0.020	0.017	0.000
Genotype x Neuroticism	0.052	0.409	0.035	0.032
*Simple Slope for A-allele carriers*				
Neuroticism	0.028	0.244	0.022	0.060
*Simple Slope for C/C genotype*				
Neuroticism	–0.024	–0.151	0.024	0.023

*The addition of robustness covariates or their interactions did not alter the significance of the primary interaction effects or the significance of simple slopes.*

### CD38 Variant rs6449182

We followed the same steps and conducted hierarchical multiple linear regression analysis and found no main or interaction effects involving CD38 rs6449182 genotype (see Table 3).

**TABLE 3:** **(a)** CD38 rs6449182 and neuroticism predicting right amygdala-sgACC functional connectivity. **(b)** CD38 rs6449182 and neuroticism predicting left amygdala-sgACC functional connectivity.

**Independent variable**	***b***	**β**	***SE***	***R*^2^**
**(a)**				
CD38 genotype	–0.011	–0.009	0.149	0.000
Neuroticism	0.023	0.145	0.019	0.021
Genotype x Neuroticism	–0.061	–0.382	0.050	0.042
*Simple Slope for G-allele carriers*				
Neuroticism	–0.027	–0.200	0.033	0.040
*Simple Slope for C/C genotype*				
Neuroticism	0.033	0.207	0.023	0.043
**(b)**				
CD38 genotype	0.095	0.083	0.138	0.007
Neuroticism	–0.001	–0.005	0.018	0.007
Genotype x Neuroticism	0.016	0.107	0.048	0.009
*Simple Slope for G-allele carriers*				
Neuroticism	0.013	0.060	0.051	0.004
*Simple Slope for C/C genotype*				
Neuroticism	–0.003	–0.026	0.018	0.001

*The addition of robustness covariates or their interactions did not alter the significance of the primary interaction effects or the significance of simple slopes.*

### Whole Brain Analyses

Full results of whole brain analyses are presented in Supplementary Tables S1, S2).

## Discussion

The present findings are the first showing evidence of a moderating role for CD38 genetic variation on the association between neuroticism and amygdala-sgACC connectivity. Specifically, there was a positive association between neuroticism and right amygdala-sgACC functional connectivity among rs3796863 A-allele carriers. Thus, A-allele carriers with lower levels of neuroticism showed more inverse functional connectivity between right amygdala and sgACC whereas A-allele carriers with higher levels of neuroticism showed more positive connectivity. For illustrative purposes, we created Supplementary Figure S1 to decompose patterns of functional connectivity. Results suggested that the present findings may be driven by A-allele carriers with lower levels of neuroticism, potentially due to better regulation of the amygdala. This finding suggests that results from our previous work, in which we found increased risk for social anxiety and depression over time among rs3796863 A-allele carriers who experienced greater interpersonal stress, may have been specific to individuals with higher levels of neuroticism, who were oversampled (Tabak et al., 2016). These results also follow the pattern shown by McQuaid et al. (2016) who found higher levels of depression and suicidal ideation among individuals with the rs3796863 AA genotype compared to C-allele carriers (but see Parris et al., 2018; Handley et al., 2019). Results also suggest that accounting for neuroticism in future studies of CD38 genetic variation may help to explain discrepant associations of the rs3796863 A-allele with outcomes such as greater empathy and altruism (Liu et al., 2017), reduced risk of autism spectrum disorders (Munesue et al., 2010), but also higher levels of depression and suicidal ideation (McQuaid et al., 2016). Since the directionality of associations among A-allele carriers has differed across studies, further research that accounts for levels of neuroticism is needed. More broadly, the present finding adds to results from previous studies suggesting a role for oxytocin system genetic variants in enhanced social sensitivity (Tabak, 2013).

The present results are also in agreement with studies showing increased connectivity between the amygdala and sgACC in individuals with depression during a facial affect recognition task for fearful stimuli (Ho et al., 2014) and among adult first-degree relatives of individuals with major depressive disorder when performing a negative affect matching task (Wackerhagen et al., 2017). In addition, a previous report found that the same neural circuit examined in the present study was moderated by genetic variation in the serotonin system (i.e. more inverse amygdala-sgACC connectivity was related to higher levels of harm avoidance among 5-HTTLPR short allele carriers; Pezawas et al., 2005). In a previous study examining the relationship between neuroticism and amygdala-anterior cingulate cortex (ACC) connectivity, Cremers et al. (2010) found that neuroticism was related to more inverse functional connectivity between the left amygdala and ACC. In the present study, our analyses did not identify a significant relationship between the left amygdala and the ACC; however, whole brain analyses showed a significant interaction effect of rs3796863 x neuroticism predicting positive functional connectivity between the right amygdala and the ACC. One potential explanation for the discrepancy between the present results and those from Cremers et al. (2010) is that the sample in the study by Cremers and colleagues included all healthy individuals, whereas our sample included healthy individuals as well as individuals with anxiety and depressive disorders.

Although previous studies have examined the role of genetic variation in 5-HTTLPR and neuroticism (Pluess et al., 2010; Kuepper et al., 2012), to date, there is limited research examining oxytocin related genetic variants and neuroticism. This seems like an important oversight since, in addition to its role in social processes, oxytocin is associated with stress responsivity (Engert et al., 2016; Alley et al., 2019) and evidence suggests that early life adversity can alter the oxytocin system (Bradley et al., 2011; Grimm et al., 2014; Smearman et al., 2016). In addition, neuroticism not only predicts psychopathology over time (Kendall et al., 2015), but it’s also associated with negative interpersonal outcomes such as increased reactivity to stressful events following conflict (Suls et al., 1998), a tendency to use negative forms of coping following interpersonal stress (Gunthert et al., 1999), and negative marital outcomes including divorce (Kelly and Conley, 1987). As studies continue to elucidate potential relationships between oxytocin and psychopathology (McQuaid et al., 2014; Gottschalk and Domschke, 2018), the present results suggest that neuroticism should be a target of future oxytocin research. This enhanced focus on neuroticism would be consistent with elevated levels of anxiety and emotional reactivity to negative events that have been seen in mice with deletion of the CD38 gene (Martucci et al., 2019).

Exploratory whole brain analyses showed main effects of neuroticism on regions that are considered part of the default mode network, such as the temporoparietal junction, precuneus, and sgACC (Menon, 2011; Li et al., 2014). These findings are consistent with prior work demonstrating altered connectivity of functional brain networks, including the default mode network, in anxiety disorders and depression (Sylvester et al., 2012; Zhu et al., 2012). Future work exploring altered network connectivity in the context of oxytocin would be of much interest in this regard. Additional whole brain analyses suggested that the interaction of genotype and neuroticism might impact other neural networks, including the ACC, dorsal medial prefrontal cortex, and inferior frontal gyrus regions. These regions have been implicated in a variety of functions including the explicit regulation of emotional reactivity in limbic brain regions (Ochsner and Gross, 2008). The current study was not designed to investigate emotion regulation, instead focusing on emotional reactivity to negative stimuli, but investigation of how neuroticism and CD38 variants interact to impact regulation of emotional reactions would be of interest in future research.

The present study has several strengths including a sample of participants with a wide range of social anxiety and depression levels, the focus on a continuous measure of psychopathology risk (i.e. neuroticism), and the examination of genetic variation of a neural circuit through functional connectivity analysis. In addition, the significant gene x neuroticism interaction effect found in the present study withstood multiple test correction and the addition of many robustness covariates and their interaction effects. However, several limitations must also be noted. Although the present sample is slightly larger than other studies examining CD38 genetic moderation of neural activation (Sauer et al., 2012, 2013), based on current recommendations (Duncan and Keller, 2011), our sample is small for a GxE interaction study. In addition, the size of the interaction effect found in the present study (*R*^2^ = 0.11 with robustness covariates; *R*^2^ = 0.217 without robustness covariates) is much larger than current estimates for typical GxE effects (Duncan and Keller, 2011). Another limitation is our racially/ethnically heterogeneous sample. To account for this in our statistical analysis, we included race/ethnicity and genotype x race/ethnicity interactions as covariates, which is an established method to statistically reduce the potential effects of population stratification (Keller, 2014). However, the size of our sample prevented us from conducting additional analyses to examine the generalizability of effects within and across racial/ethnic subgroups. Based on these limitations, replication studies with a larger sample size are necessary, and the present results should be viewed as preliminary in nature.

There is evidence that CD38 gene expression is positively associated with levels of endogenous oxytocin (Kiss et al., 2011), but the way in which CD38 SNP rs3796863 may influence genetic expression is not yet known. Therefore, the present findings suggest that rs3796863 may be tagging a functional SNP that was not genotyped in our study (Lin et al., 2007). In contrast, several studies have found evidence for a functional role for rs6449182 (Jamroziak et al., 2009; Polzonetti et al., 2012), but variation in this SNP was not associated with our outcome. The present study also did not include a direct measurement of endogenous oxytocin, which precludes us from examining the relationship between CD38 genetic variation, circulating levels of oxytocin, and neuroticism. However, previous work has found an association between CD38 genetic variation and differences in levels of unextracted oxytocin (Feldman et al., 2012).

### Conclusion

In sum, we found a positive association between neuroticism and right amygdala-sgACC functional connectivity in rs3796863 A-allele carriers. Given the correlational nature of functional connectivity analysis, the extent to which the right amygdala is affecting the sgACC or vice versa cannot be determined. However, the present results suggest that the more socially sensitive rs3796863 A-allele may partially explain the relationship between a known risk factor (i.e. neuroticism) and promising biomarker (i.e. amygdala-sgACC connectivity) in the development and maintenance of social anxiety and depression.

## Data Availability Statement

The datasets for this manuscript are not publicly available because consent was not obtained from participants for this purpose during the randomized controlled trial from which this data came (Craske et al., 2014). Requests to access the datasets should be directed to MC, MCraske@mednet.ucla.edu.

## Ethics Statement

The studies involving human participants were reviewed and approved by the UCLA Office for the Protection of Human Research Subjects and the UCLA Institutional Review Board. The patients/participants provided their written informed consent to participate in this study.

## Author Contributions

LB, ML, and MC designed the original study. BT conceptualized the present study. BT, KY, JT, and BW analyzed the data. BT and KY wrote the first draft of the manuscript. BT, KY, BW, LB, NE, ML, and MC contributed to the manuscript revision, read, and approved the submitted version.

## Conflict of Interest

The authors declare that the research was conducted in the absence of any commercial or financial relationships that could be construed as a potential conflict of interest.

## Footnotes

1 www.Brainmapping.org/WhiteAnt

2 http://www.fil.ion.ucl.ac.uk
