# Supplementary material for: Preliminary Evidence That CD38 Moderates the Association of Neuroticism on Amygdala-Subgenual Cingulate Connectivity
Source: Front Neurosci. 2020 Feb 14;14:11. doi: 10.3389/fnins.2020.00011 (PMC7033443; doi:10.3389/fnins.2020.00011)
Supplement: Supplementary file 1 [file Data_Sheet_1.PDF]

Figure S1.
CD38 rs3796863 x neuroticism predicting right amygdala-sgACC functional connectivity for affect match and shape match separately

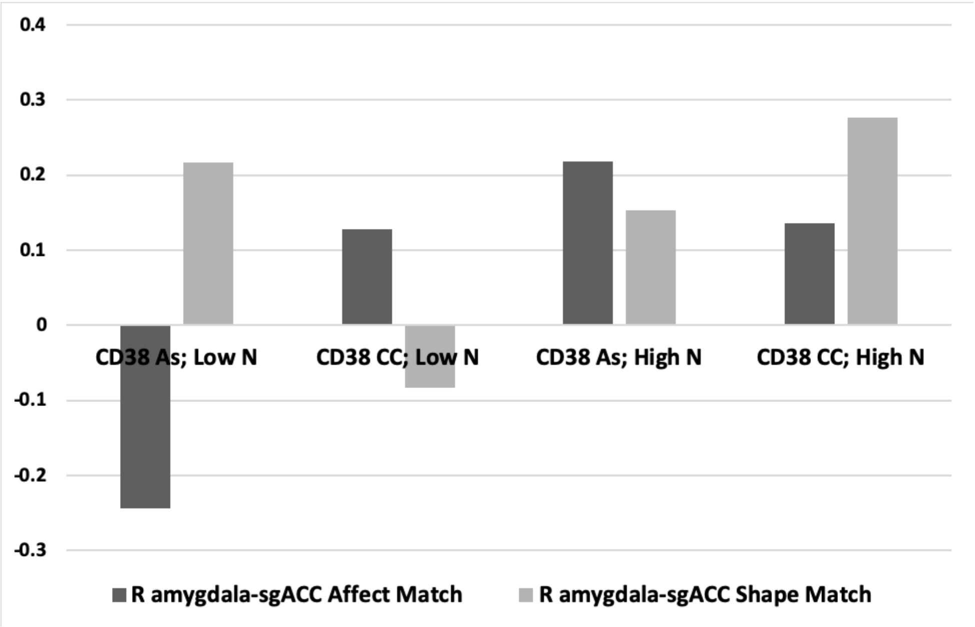

Note. This figure is meant for illustrative purposes to decompose patterns of functional connectivity. Low and high levels of neuroticism are based on +1 and -1 SD from the mean level of neuroticism. Participants who are not represented in either category are not represented in this figure. CD38 As= rs3796863 A-allele carriers; CD38 CC= rs3796863 CC genotype; R amygdala = right amygdala; sgACC=subgenual anterior cingulate cortex; N=neuroticism.

Table S1.
Results of whole brain connectivity analyses with right amygdala seed region using a p-value of .005 combined with an extent threshold of 40 contiguous voxels

**Anatomical region**	**Hem**	**Brodmann's Area**	**MNI peak coordinates**			***t***	***k***
			**x**	**y**	**z**		
rs3796863 main effect							
Postcentral gyrus	L	3	-60	-19	37	4.70	320
Superior temporal gyrus	L	41	-51	-25	4	4.00	320
Superior temporal gyrus	L	22	-60	-37	19	3.89	320
Postcentral gyrus	R	3	63	-16	19	4.28	240
Superior temporal gyrus	R	41	45	-28	16	4.17	240
Superior temporal gyrus	R	22	66	-40	13	4.15	240
Paracentral motor area	R	5	0	-37	61	4.05	64
Supplemental motor area	R	6	12	-22	58	3.35	64
Neuroticism main effect							
Subgenual ACC	Bi	32	-3	38	-11	5.18	93
Orbitofrontal cortex	L	11	-18	44	-11	-5.38	42
Inferior parietal lobe	R	7	21	-64	34	-3.62	55
Temporoparietal junction	R	39	33	-58	28	-2.92	55
Precuneus	L	7	-6	-67	43	-3.18	42
Precuneus	L	7	-15	-70	34	-2.82	42
rs3796863 x neuroticism interaction effect							
Anterior cingulate cortex	Bi	24	3	38	1	4.34	78
Medial prefrontal cortex	R	10	6	53	-5	3.75	78
Midcingulate	Bi	32	0	17	34	4.19	43
Dorsal medial prefrontal cortex	Bi	9	0	32	34	3.13	43
Temporoparietal junction	L	40	-60	-49	28	3.77	69
Middle temporal gyrus	L	22	-45	-55	16	3.17	69
Supramarginal gyrus	L	40	-51	-52	25	3.17	69
Inferior frontal gyrus (triangularis)	L	45	51	20	19	3.68	57
Inferior frontal gyrus (triangularis)	L	46	42	20	28	3.15	57
Inferior frontal gyrus (triangularis)	L	46	51	32	22	3.05	57

Table S2.
Results of whole brain connectivity analyses with left amygdala seed region using a p-value of .005 combined with an extent threshold of 40 contiguous voxels

**Anatomical region**	**Hem**	**Brodmann's Area**	**MNI peak coordinates**			***t***	***k***
			**x**	**y**	**z**		
rs3796863 main effect							
Inferior Frontal Gyrus	L	47	-48	23	-5	4.01	118
Orbitofrontal Gyrus	L	11	-45	32	-8	3.89	118
Postcentral Gyrus	L	40	54	-34	52	3.87	101
Postcentral Gyrus	R	2	42	-34	58	3.23	101
Supramarginal Gyrus	R	40	42	-55	40	3.14	101
Postcentral Gyrus	R	6	63	-16	40	3.66	107
Postcentral Gyrus	R	6	63	-7	34	3.59	107
Parietal Operculum	R	43	54	-13	22	3.16	107
Middle Temporal Gyrus	R	39	54	-67	22	3.62	44
Angular Gyrus	R	39	42	-76	31	3.18	44
Superior Occipital Gyrus	R	19	33	-79	31	3.04	44
Cuneus	R	18	9	-88	16	3.62	100
Calcarine	R	17	6	-82	1	3.34	100
Lingual Gyrus	L	18	-3	-79	-8	2.91	100
Inferior Parietal Lobe	L	40	-42	-55	55	3.61	212
Supramarginal Gyrus	L	40	-42	-52	37	3.53	212
Angular Gyrus	L	7	-36	-70	43	3.48	212
Middle Temporal Gyrus	L	20	-54	-40	-8	3.43	45
Superior Temporal Gyrus	L	22	-48	-22	1	3.27	45
Inferior Temporal Gyrus	L	37	-57	-52	-14	3.15	45
rs3796863 x neuroticism interaction effect							
Calcarine	L	30	-12	-67	4	3.8	47
